# Supplementary material for: Patterns of Intron Gain and Loss in Fungi
Source: PLoS Biol. 2004 Nov 30;2(12):e422. doi: 10.1371/journal.pbio.0020422 (PMC532390; doi:10.1371/journal.pbio.0020422)
Supplement: Table S1 — Also available at http://genes.mit.edu/NielsenEtAl/. (4.3 MB ZIP). [file pbio.0020422.st001.zip › NielsenEtAl/html/770.html]

AN8204.1.NCU00293.1.MG04697.1.FG05580.1


```
 CLUSTAL W (1.82) Multiple Sequence Alignments - Introns Inserted


Sequence 1: MG04697.1	481 aa
Sequence 2: FG05580.1	416 aa
Sequence 3: NCU00293.1	433 aa
Sequence 4: AN8204.1	415 aa
Alignment Length: 538 aa
Number Identitical Residues: 121 aa
Alignment Score (without introns) 7166


MG04697.1 	MQATKWTCINFIGLDAT2LAASTPLFWKDQSCR1IAGAGIDSLRKRSRRQSTSRPHHTIP
NCU00293.1	-----------------~---------------~--------------------------
FG05580.1 	-----------------~---------------~--------------------------
AN8204.1  	-----------------~---------------~--------------------------
          	                                                            

MG04697.1 	KTPILLPRSPTRSPLNVPNLVEPSTPGLLQQQKDTRFECATKMAPSPLLPQLPADDSMLT
NCU00293.1	-------------------------------------MPPPALPTLSYLSQ-ESGASALT
FG05580.1 	------------------------------------MSSDTAAPNLPRVAALENDDSMLT
AN8204.1  	------------------------------------MSNNSQIPNPAHIQA----DSMLA
          	                                        .  .  . :       * *:

MG04697.1 	RRFGAETANYFSG0NPLNRVSFLRDNRDFLQAAFAHPSARFLALDKLAPLVHTAEKS--I
NCU00293.1	RKFGPETANYFSG~SPLNRLSWLRSDHLFLRAAFSHASARFLLMNNLGPMVESKQNDGRL
FG05580.1 	RRFGKEVVNYYAG~SRINRFSFLRADTGFLRKAAASPTARYLALSELNPLV-VDKRT--P
AN8204.1  	RRFGKETVNYFSS1SPLNRLSFLRSDHPFLSAAFKHPSTRFVILKDLAPLTRSPTEL---
          	*:** *..**::. . :**.*:** :  **  *   .::*:: :..* *:.    .    

MG04697.1 	AWITREDVAPVTGSGP-FDKTEEDMINDFNSEESHP----VIILLG--LDDKQKMTAS--
NCU00293.1	AFAGPEDVRALLGSGEEVFRSEEEVVGGYDSEKAAGKGERMVVFLGVDLVDEKKQQQPQG
FG05580.1 	AYLTFNDVEPLIGSDP-FAQTEDEAIQNFDSTKTTP----LIVFLG--MLEEGNEND---
AN8204.1  	YYAKYDEVRKLLPASI-YDKSEEEIIKEHDSRKTEP----HLIFLG---VDETDKQQ---
          	 :   ::*  :  :.    ::*:: :  .:* ::       :::**    :: .      

MG04697.1 	-----DQDVFQHKDYKGAPYFAVDFT----------ENQDVVTSLASREG--LSFNQDQR
NCU00293.1	QGQEGGEDVFVWKEFRGAPYFAVDVTPREGDGEEGKAKAEELIKKMEEEKGHAFLGASAR
FG05580.1 	-----HISSTDHGDILGHPYFAIDITPKGNHA----EKAESFLAEQEKEE--MSLDKNPR
AN8204.1  	-------DGFSWKIYSGTPYFALDVSEKGSEEQQ--HNAKEVVNGFEAQG--LSFLQARV
          	       .        * ****:*.:   .  ..   : . .    . :     :     

MG04697.1 	SLGFNPGEE1DITGPRRSKTRLS-------------------------PTDMA--GGEKR
NCU00293.1	GMALEAGHA1AMYGQARAVVDWNARNPFCAQCGQRTISVHAGTKRVCPPTDK----GKD-
FG05580.1 	AMGHSPEAA1ALYAQARSMMDWNSRSPFCAGCGQPNLSVHAGYKRVCPPADKK--GGENS
AN8204.1  	AMTFSANEA1AIYAQARALIDWNTRNSYCGTCGSRTISVNSGTKRACPPTDAARVAEGKP
          	.:  ..     : .  *:    .: .. .. ... . :  :. .  ..*:*    .  . 

MG04697.1 	DRK-PCASRGTVSNISFPRTDPTIIVAVVSADGTKVLLGRNKRWPKYWYSTLAGFLEPGE
NCU00293.1	--RAPCATRGTVSNLSFPRTDPTVIMAIINADGTKVLLGRNRRWPQYWYSTLAGFQEPGE
FG05580.1 	EARGDCATRHGVSNICFPRTDPTMIAAVVSADGTKILLGRQKRWPPYWYSTLAGFLEPGE
AN8204.1  	AEKPACSTRTTISNLSFPRTDPTIIVAVISSDAKRILLGRSKRFPPNWYSTLAGFIEPAE
          	  :  *::*  :**:.*******:* *::.:*..::****.:*:*  ******** **.*

MG04697.1 	SIEEATRREVWEESGVTVGRVVLHSSQPWPFPGSLMIGAIAQALPGDGEKIFLGNDPELE
NCU00293.1	SIEEAVRREVHEESGVKVGRVVLHSSQPWPFPASLMIGAIGQALPGDGEKIFLGHDAELE
FG05580.1 	SIEESVRREVWEESGVRVGRVVIHSSQPWPYPASLMIGAIAQALPGDGENITL-NDKELE
AN8204.1  	SVEDAVRREVWEEAGVTLSRVIIHSTQPWPYPANLMIGAIAQVSDPAHEKINLEHDPELE
          	*:*::.**** **:** :.**::**:****:*..******.*.     *:* * :* ***

MG04697.1 	DAKWFPMAEVRKALSSGVSNLGQSAPPEYVEGDLRVPPDTAIANRLMTAVVGG-YNGAFP
NCU00293.1	DAKWFPFEEVKEALLNGVSALGEAAPAGYVEGALRLPPQTAIANRLVKAVVEG-WWTMS-
FG05580.1 	AAKWFTLEEARKALQSGTSSLGASAPEGYKEGDLRLPPSQAIANRLITAVVEG-YLVVAP
AN8204.1  	DARWFEIEEVEEALRVGTSNLDQAAGPGYKEGSLRLPPPTAIANQLIQAAINIDFLADKS
          	 *:** : *..:**  *.* *. :*   * ** **:**  ****:*: *.:  .:    .

MG04697.1 	KI
NCU00293.1	KI
FG05580.1 	KI
AN8204.1  	KM
          	*:
```
